# Efficacy and Safety of Direct Oral Anticoagulant for Treatment of Atrial Fibrillation in Cerebral Amyloid Angiopathy

**DOI:** 10.7759/cureus.10143

**Published:** 2020-08-30

**Authors:** Koichi Narita, Eisuke Amiya, Issei Komuro

**Affiliations:** 1 Department of Cardiovascular Medicine, The University of Tokyo, Tokyo, JPN; 2 Department of Therapeutic Strategy for Heart Failure, The University of Tokyo, Tokyo, JPN

**Keywords:** cerebral amyloid angiopathy, atrial fibrillation, anticoagulant therapy, direct oral anticoagulant, oxidative stress

## Abstract

A 75-year-old man with a history of atrial fibrillation (AF) and anticoagulant therapy presented with a headache. Cerebral amyloid angiopathy (CAA) was diagnosed after MRI of the brain revealed cortical superficial siderosis, lobar intracerebral hemorrhage, and lobar microbleeds. Anticoagulant therapy was carefully discontinued. Several years later, he was admitted with sudden onset left upper-extremity weakness. In addition to CAA bleeding lesions, a diffusion-weighted brain MRI showed multiple infarct lesions of high signal intensity. The administration of edoxaban 7.5 mg/day (later increased up to 30 mg/day) prevented ischemic stroke recurrence without exacerbation of cerebral bleeding. This could indicate that CAA patients with AF who had previous adverse effects from warfarin can safely use newer direct oral anticoagulants, such as edoxaban, to prevent ischemic stroke without danger of cerebral hemorrhage. The superiority of edoxaban as compared with warfarin might be due to its antioxidant effect because vascular oxidative stress plays a causal role in CAA-induced cerebrovascular dysfunction, CAA-induced cerebral hemorrhage, and CAA formation itself. We explained the beneficial effect of edoxaban for CAA by the mechanism of oxidative stress in the paper.

## Introduction

Atrial fibrillation (AF) is increasing in prevalence with aging, and the main cause of cerebral infarction is AF. The anticoagulant therapy is effective in reducing the risk of cerebrovascular infarction in AF [1,2]; however, there are several situations in which the balance of risk and benefit of this strategy has much difficulties. Indeed, the risk of bleeding due to anticoagulant therapy is also critical [3,4]. Therefore, it is important to evaluate the risk-benefit of anticoagulant therapy.

Cerebral amyloid angiopathy (CAA) is caused by the accumulation of β-amyloid (amyloid β40 proteins) in the cerebral arteries [5]. CAA is associated with intracerebral hemorrhage and the risk of recurrent bleeding is increased [6,7]. In addition, the risk of bleeding is presumed to be significantly enhanced in the treatment of anticoagulant therapy. On the other hand, direct oral anticoagulants (DOAC), such as edoxaban, have been shown to have a lower risk of cerebral bleeding as compared with warfarin in previous clinical trials [8]. However, the safety of DOAC for ischemic stroke in CAA patients with AF has not been obvious. We describe a CAA patient with ischemic stroke caused by AF in whom DOAC may be alternative to warfarin for the prevention of ischemic stroke without cerebral bleeding event.

## Case presentation

A 75-year-old man admitted to our outpatient clinic with an irregular pulse was diagnosed with AF by electrocardiogram. He had hypertension, diabetes, and previous stroke and scored 5 on the CHA2DS2-VASc (congestive heart failure, hypertension, age ≥ 75 years, diabetes mellitus, stroke or transient ischemic attack, vascular disease, age 65 to 74 years, sex category) assessment of AF and stroke; treatment with vitamin K antagonist oral anticoagulant (warfarin) was initiated [9].

**Figure 1:**
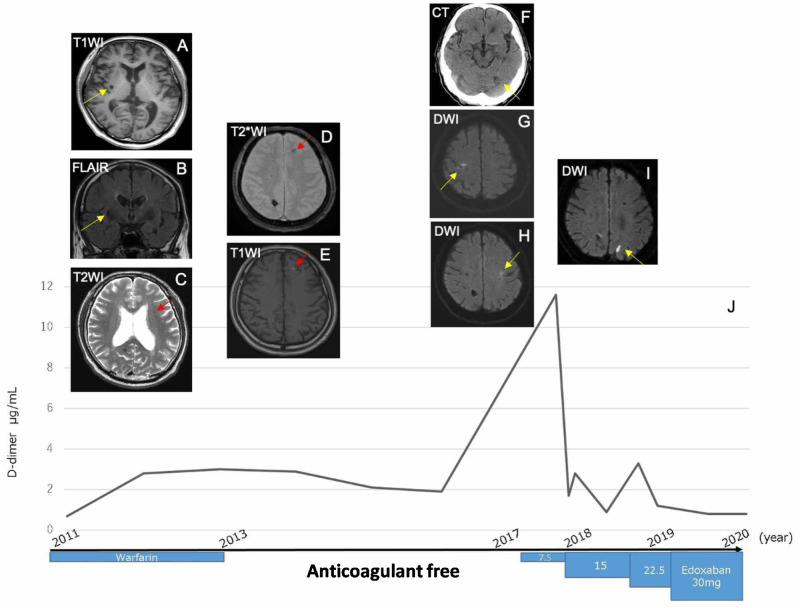
Summary of the progress of this case A: T1-weighted image (T1WI) sequence showing a hypodensity lesion in the right putamen. B: Axial fluid-attenuated inversion recovery (FLAIR) sequences showing an isodensity with 7 mm at the same lesion with A, which suggested old cerebral bleeding. C: T2WI sequence showing hemosiderosis in the left frontal lobe. D: T2*WI sequence showing a low-intensity lesion in the left frontal lobe. E: T1WI sequence showing high intensities in the same lesion as D, in the left frontal lobe, which suggests subacute cerebral hemorrhage. F: Cerebral CT showing left cerebellar infarction. G, H: Diffusion-weighted image (DWI) sequence showing multiple deep and cortical infarct lesions, including right central groove lesion. I: DWI sequence showing recurrence of cerebral infarction in other sites. J: Time course of the level of D-dimer and medical treatment about anticoagulation.

A few months later, he presented with a headache, and CAA was suspected after a brain MRI showed multiple hemorrhages in the cortical regions and cortical superficial siderosis with ischemic changes (Figure 1A-1C). We carefully adjusted and lowered the doses of warfarin while measuring the prothrombin time international normalized ratio (PT-INR). A follow-up MRI showed a recurrent cerebral hemorrhage due to CAA (Figure 1D, 1E), and anticoagulant therapy was discontinued.

A few years after discontinuing warfarin, he was admitted with sudden-onset difficulties with left-hand movement, and a cerebral CT showed a cerebellar infarction (Figure 1F). A diffusion-weighted brain MRI showed other multiple new lesions of high signal intensity (Figure 1G, 1H), but no thrombus was seen in a transesophageal echocardiogram. To prevent a thrombotic stroke recurrence, however, we resumed anticoagulant therapy with a very small amount of edoxaban, gradually increasing the dose to 30 mg/day and monitoring the value of the D-dimer. There was one minor event of cerebral infarction before the dose increase (Figure 1I), but no events of cerebral hemorrhage and infarction have occurred in the several years since the edoxaban was initiated (Figure 1J).

## Discussion

We describe a CAA patient with ischemic stroke caused by AF, which increases in prevalence with age and is the primary cause of cerebral infarction [10]. Anticoagulant therapy reduces the risk of cerebrovascular infarction in AF, but the benefits and risks of this strategy should be weighed in cases of hemorrhagic diathesis. Patients with CAA, which results from vascular injury by the accumulation of β-amyloid in the cerebral arteries, have a high risk of repeated intracerebral hemorrhage [11].

The mechanism of bleeding in CAA had not been concisely understood. Gurol et al. demonstrated the link between vascular amyloid burden and risk of bleeding [12]. In addition, the deposition of β-amyloid in the cerebrovascular export pathway resulted in further damage to blood vessels and aggravated CAA [13]. However, the risk of bleeding could not be predicted; therefore, there had been no helpful information about the decision about anticoagulant therapy, including warfarin [14]. There were some complex mechanisms relating oxidative stress underlying the mechanism of the progression of CAA. Han et al. demonstrated reactive oxygen species (ROS) and, in particular, nicotinamide adenine dinucleotide phosphate (NADPH) oxidase-derived ROS are a key mediator of CAA-induced vascular dysfunction and anti-ROS therapy attenuates CAA-related microhemorrhage [15]. Indeed, there were some reports about the anti-ROS effect of edoxaban. Narita et al. reported that edoxaban exerted antioxidant effects through factor Xa inhibition and direct radical-scavenging activity using human proximal tubular cells [16].

Although our patient had adverse bleeding events with warfarin several years earlier, he was successfully treated with edoxaban [17,18]. Rates of subdural and subarachnoid hemorrhage seem to be lower with DOAC than with warfarin [19]. Additionally, reactive oxygen stress is thought to play a role in CAA. The superiority of edoxaban in CAA might be due to its anti-ROS effects, which should be further explored in a large clinical studies [20].

## Conclusions

We report the efficacy and safety of DOAC for the CAA with AF. Based on the experience of this case, early anticoagulant therapy with DOAC may be more appropriate than warfarin in coexistence case of CAA and AF.
